# Supplementary material for: The Systems Biology Research Tool: evolvable open-source software
Source: BMC Syst Biol. 2008 Jun 29;2:55. doi: 10.1186/1752-0509-2-55 (PMC2446383; doi:10.1186/1752-0509-2-55)
Supplement: Additional file 1 — SBRT Archive. An archive of the current version of the Systems Biology Research Tool. [file 1752-0509-2-55-S1.zip › sbrt-1.4.0/doc/developers_guide/api/sbrt/kernel/util/Util.html]

Util


|  |  |  |
| --- | --- | --- |
| **Overview** \| **Package** \| **Class** \| **Use** \| **Tree** \| **Deprecated** \| **Index** \| **Help** | |  |
| **PREV CLASS**   NEXT CLASS | **FRAMES**    **NO FRAMES**     **All Classes** |  |
| SUMMARY: NESTED \| FIELD \| CONSTR \| METHOD | DETAIL: FIELD \| CONSTR \| METHOD |  |


---


## sbrt.kernel.util Class Util

```
java.lang.Object
  sbrt.kernel.util.Util
```

---

``` public final class Util extends java.lang.Object ```

This class contains static methods that perform utility functions.

**Author:**
:   This class was written and documented by Jeremiah Wright
    while in the Wagner lab.

---

| **Method Summary** | |
| --- | --- |
| `static void` | `debugPrintln(java.lang.Object o)`             Prints the provided object to `System.err`, preceded by a carriage return character. |
| `static boolean` | `equal(double value1, double value2, double tolerance)`             Indicates if the provided values are equal to each other, given the provided tolerance. |
| `static double[]` | `getArray(java.util.Collection<? extends java.lang.Number> values)`             Returns an array containing the values in the provided collection as doubles. |
| `static int[]` | `getArray(java.util.Collection<java.lang.Integer> values)`             Returns an array containing the values in the provided collection. |
| `static double[][]` | `getArrays(java.util.Map<?,java.lang.Double> xValues, java.util.Map<?,java.lang.Double> yValues)`             Returns a `2 x n` array containing the values from the provided maps. |
| `static <E> java.util.List<E>` | `getCommonElements(java.util.Collection<? extends E> c1, java.util.Collection<? extends E> c2)`             Returns a list of elements common to both provided collections. |
| `static <E> java.util.List<E>` | `getDuplicates(java.util.Collection<E> c)`             Returns a list of duplicate elements contained in the provided collection. |
| `static double` | `getLargest(double v1, double v2)`             Returns the largest of the provided numbers. |
| `static int` | `getLargest(int v1, int v2)`             Returns the largest of the provided numbers. |
| `static double` | `getSmallest(double v1, double v2)`             Returns the smallest of the provided numbers. |
| `static int` | `getSmallest(int v1, int v2)`             Returns the smallest of the provided numbers. |
| `static double[]` | `getSorted(double[] values)`             Returns a new array containing the values from the provided array in ascending numerical order. |
| `static int[]` | `getSorted(int[] values)`             Returns a new array containing the values from the provided array in ascending numerical order. |
| `static double[]` | `getSortedArray(java.util.Collection<? extends java.lang.Number> values)`             Returns a sorted array containing the values in the provided collection. |
| `static int[]` | `getSortedArray(java.util.Collection<java.lang.Integer> values)`             Returns a sorted array containing the values in the provided collection. |
| `static boolean` | `isZero(double d)`             Indicates if the provided value equals 0. |
| `static void` | `main(java.lang.String[] args)`             Used for testing purposes. |
| `static void` | `nullCheck(java.lang.Object obj)`             Ensures the provided object is not null. |
| `static boolean` | `oppositeSign(double x1, double x2)`             Indicates if the provided numbers are of opposite sign. |
| `static boolean` | `oppositeSign(int x1, int x2)`             Indicates if the provided numbers are of opposite sign. |
| `static void` | `print(java.util.Collection<?> c)`             Prints the elements of the provided collection to `System.out`, one per line. |
| `static java.lang.String` | `widthFormat(java.lang.String string, int maxWidth)`             Attemps to format the provided string such that system-dependent newline characters appear no more than `maxWidth` characters apart. |

**Methods inherited from class java.lang.Object**

`clone, equals, finalize, getClass, hashCode, notify, notifyAll, toString, wait, wait, wait`

**Method Detail**

### nullCheck

```
public static void nullCheck(java.lang.Object obj)
```

:   Ensures the provided object is not null.

    :   **Parameters:**: `obj` - the object to check. **Throws:**: `java.lang.NullPointerException` - if the provided argument is `null`.

---


### getSorted

```
public static int[] getSorted(int[] values)
```

:   Returns a new array containing the values from the provided array
    in ascending numerical order.
    The returned array is completely autonomous relative to the provided array.

    :   **Parameters:**: `values` - the array whose values are to be sorted. **Returns:**: a new array containing the values from the provided array in ascending numerical order.

---


### getSorted

```
public static double[] getSorted(double[] values)
```

:   Returns a new array containing the values from the provided array
    in ascending numerical order.
    The returned array is completely autonomous relative to the provided array.

    :   **Parameters:**: `values` - the array whose values are to be sorted. **Returns:**: a new array containing the values from the provided array in ascending numerical order.

---


### getArray

```
public static double[] getArray(java.util.Collection<? extends java.lang.Number> values)
```

:   Returns an array containing the values in the provided collection as doubles.
    The order of appearance of values in the array is the same as that
    returned by an iterator over the collection.
    The returned array is completely autonomous relative to the provided collection.

    :   **Parameters:**: `values` - a collection of double precision values. **Returns:**: an array containing the values in the provided collection. **See Also:**: `Number.doubleValue()`

---


### getArray

```
public static int[] getArray(java.util.Collection<java.lang.Integer> values)
```

:   Returns an array containing the values in the provided collection.
    The order of appearance of values in the array is the same as that
    returned by an iterator over the collection.
    The returned array is completely autonomous relative to the provided collection.

    :   **Parameters:**: `values` - a collection of double precision values. **Returns:**: an array containing the values in the provided collection.

---


### getSortedArray

```
public static double[] getSortedArray(java.util.Collection<? extends java.lang.Number> values)
```

:   Returns a sorted array containing the values in the provided collection.
    The returned array is completely autonomous relative to the provided collection.

    :   **Parameters:**: `values` - a collection of double precision values. **Returns:**: an array containing the values in the provided collection. **See Also:**: `Arrays.sort(double[])`, `Number.doubleValue()`

---


### getSortedArray

```
public static int[] getSortedArray(java.util.Collection<java.lang.Integer> values)
```

:   Returns a sorted array containing the values in the provided collection.
    The returned array is completely autonomous relative to the provided collection.

    :   **Parameters:**: `values` - a collection of double precision values. **Returns:**: an array containing the values in the provided collection. **See Also:**: `Arrays.sort(int[])`

---


### getArrays

```
public static double[][] getArrays(java.util.Map<?,java.lang.Double> xValues,
                                   java.util.Map<?,java.lang.Double> yValues)
```

:   Returns a `2 x n` array containing the values from the provided
    maps. The array at index `0` contains the x values, and the array
    at index `1` contains the y values. The order of appearance of
    values in these arrays is the same as that returned by an iterator over
    `xValues.entrySet()`. The key sets of the provided maps need not
    be identical, but only the intersecting values will be considered when
    filling the arrays. The lengths `n` of these arrays equals the size
    of the intersection of the key sets of the provided maps. Therefore, if
    the provided maps contain identical keys, then `n == xValues.size() &&
    n == yValues.size()` evaluates to `true`.

    :   **Parameters:**: `xValues` - keys mapped to values.: `yValues` - keys mapped to values. **Returns:**: a `2 x n` array containing the values from the provided maps.

---


### isZero

```
public static boolean isZero(double d)
```

:   Indicates if the provided value equals 0.

    :   **Parameters:**: `d` - the value to test. **Returns:**: `Math.abs(d) < Double.MIN_VALUE;`.

---


### equal

```
public static boolean equal(double value1,
                            double value2,
                            double tolerance)
```

:   Indicates if the provided values are equal to each other, given the
    provided tolerance.

    :   **Parameters:**: `value1` - a value.: `value2` - a value.: `tolerance` - the amount by which the provided values can differ but still be considered equal. **Returns:**: `Math.abs(value1 - value2) < tolerance;`

---


### widthFormat

```
public static java.lang.String widthFormat(java.lang.String string,
                                           int maxWidth)
```

:   Attemps to format the provided string such that system-dependent
    newline characters appear no more than `maxWidth` characters
    apart. This method is not robust.

    :   **Parameters:**: `string` - the string to format.: `maxWidth` - the ideal maximum distance between newline characters. **Returns:**: the formatted version of the provided string.

---


### debugPrintln

```
public static void debugPrintln(java.lang.Object o)
```

:   Prints the provided object to `System.err`, preceded by a carriage
    return character.

    :   **Parameters:**: `o` - the object to print.

---


### print

```
public static void print(java.util.Collection<?> c)
```

:   Prints the elements of the provided collection to `System.out`,
    one per line.

    :   **Parameters:**: `c` - the collection to be printed.

---


### getSmallest

```
public static double getSmallest(double v1,
                                 double v2)
```

:   Returns the smallest of the provided numbers.

    :   **Parameters:**: `v1` - a numerical value.: `v2` - a numerical value. **Returns:**: the smallest of the provided numbers.

---


### getLargest

```
public static double getLargest(double v1,
                                double v2)
```

:   Returns the largest of the provided numbers.

    :   **Parameters:**: `v1` - a numerical value.: `v2` - a numerical value. **Returns:**: the largest of the provided numbers.

---


### getSmallest

```
public static int getSmallest(int v1,
                              int v2)
```

:   Returns the smallest of the provided numbers.

    :   **Parameters:**: `v1` - an integer value.: `v2` - an integer value. **Returns:**: the smallest of the provided numbers.

---


### getLargest

```
public static int getLargest(int v1,
                             int v2)
```

:   Returns the largest of the provided numbers.

    :   **Parameters:**: `v1` - an integer value.: `v2` - an integer value. **Returns:**: the largest of the provided numbers.

---


### getDuplicates

```
public static <E> java.util.List<E> getDuplicates(java.util.Collection<E> c)
```

:   Returns a list of duplicate elements contained in the provided
    collection.

    :   **Type Parameters:**: `E` - the element type. **Parameters:**: `c` - a collection of elements. **Returns:**: a list of duplicate elements contained in the provided collection.

---


### getCommonElements

```
public static <E> java.util.List<E> getCommonElements(java.util.Collection<? extends E> c1,
                                                      java.util.Collection<? extends E> c2)
```

:   Returns a list of elements common to both provided collections.

    :   **Type Parameters:**: `E` - the element type. **Parameters:**: `c1` - a collection of elements.: `c2` - a collection of elements. **Returns:**: a list of elements common to both provided collections.

---


### oppositeSign

```
public static boolean oppositeSign(double x1,
                                   double x2)
```

:   Indicates if the provided numbers are of opposite sign.

    :   **Parameters:**: `x1` - a numerical value.: `x2` - a numerical value. **Returns:**: `true` if one number is positive and the other negative; `false` otherwise.

---


### oppositeSign

```
public static boolean oppositeSign(int x1,
                                   int x2)
```

:   Indicates if the provided numbers are of opposite sign.

    :   **Parameters:**: `x1` - an integer value.: `x2` - an integer value. **Returns:**: `true` if one number is positive and the other negative; `false` otherwise.

---


### main

```
public static void main(java.lang.String[] args)
```

:   Used for testing purposes.

    :   **Parameters:**: `args` - ignored.


---


|  |  |  |
| --- | --- | --- |
| **Overview** \| **Package** \| **Class** \| **Use** \| **Tree** \| **Deprecated** \| **Index** \| **Help** | |  |
| **PREV CLASS**   NEXT CLASS | **FRAMES**    **NO FRAMES**     **All Classes** |  |
| SUMMARY: NESTED \| FIELD \| CONSTR \| METHOD | DETAIL: FIELD \| CONSTR \| METHOD |  |


---
